# Israeli Druze women’s sex preferences when choosing obstetricians and gynecologists

**DOI:** 10.1186/s13584-015-0013-z

**Published:** 2015-06-01

**Authors:** Jonia Amer-Alshiek, Tahani Alshiek, Yifat Amir Levy, Foad Azem, Ami Amit, Hadar Amir

**Affiliations:** Department of Obstetrics and Gynecology, Sara Racine IVF Unit, Lis Maternity Hospital, Tel Aviv Sourasky Medical Center, 6 Weizman Street, Tel Aviv, 6423906 Israel; The Faculty of Medicine, Hebrew University, Jerusalem, Israel; Neuroimmunology Laboratory, Department of Neurology, Tel Aviv Sourasky Medical Center, Sackler Faculty of Medicine, Tel-Aviv University, Tel-Aviv, Israel; Departments of Medicine, University of California, La Jolla, San Diego, CA USA

**Keywords:** Druze, Religious, Obstetricians/gynecologists, Sex

## Abstract

**Background:**

Consideration and better understanding of patients’ needs on the part of the healthcare system might help increase the number of people seeking necessary medical care. Many studies have been conducted on patients’ preferences in choosing their health care provider, but the majority of them were conducted in modern western societies, establishing a need to explore other populations. The present study was performed in the Israeli Druze community which is composed of a uniquely traditional and religious population.

We assessed the sex preference of Israeli Druze women regarding obstetricians/gynecologists, and identify other features that affect their choice.

**Method:**

We conducted a cross-sectional study that included 196 Israeli Druze women who anonymously completed a 36-item questionnaire between January-July, 2011.

**Results:**

Most (63.8%) of the responders preferred female obstetricians/gynecologists, while 74.5% had no sex preference for their family physicians. 68.6% of the religious women preferred female obstetricians/gynecologists as compared to 51.76% of those women who self-identified as secular. Most of the women (65%) preferred female obstetricians/gynecologists for intimate procedures, such as pelvic examination and pregnancy follow-up. The main reasons given were: feeling more comfortable with a female practitioner (69.7%), the belief that females are more gentle (56.6%), and being more embarrassed with male obstetricians/gynecologists (45.4%). Three factors were associated with the responders’ preferences for female obstetricians/gynecologists: their age and religious status, and the sex of their regular obstetricians/gynecologists. Women who preferred a female obstetrician/gynecologist assigned a lesser weight to the physician’s knowledge when choosing them. Older and religious women as well as those who attributed less weight to the physician’s professional knowledge were more likely to prefer a female obstetrician/gynecologist.

**Conclusions:**

The majority of responders to our survey (Israeli Druze women), like those in other communities where religiousness and modesty are deeply rooted, prefer female obstetricians/gynecologists, with the overwhelming reasons given being feeling more comfortable and less embarrassed with females, and the notion that female obstetricians/gynecologists are more gentle during intimate procedures.

## Background

Consideration and better understanding of the patients’ needs on the part of the healthcare system might help increase the number of people seeking necessary medical care. Many researchers have investigated factors that are important to women when choosing their health care provider. One of those factors is sex preference, which is likely to have a stronger impact when choosing health professionals engaged in intimate and psychosocial medical practices. Indeed, many studies have found that women prefer female physicians, especially when it comes to obstetrical/gynecological issues [1-4]. In 2004, Sampietro-Colom et al. noted that although there are many publications regarding same sex preference, it is limited to a fairly homogeneous population (Caucasian women from the United States, the United Kingdom, and Canada) [5]. Several recent publications have discussed the preferences of Arab women [6-8], Asian women [9], and Israeli women [10-13], and many explanations were given for their preferring same-sex physicians, among them the nature of the required clinical care, the physician’s attitude and communication style, and the patient’s stereotyping of physicians [14,15]. Notably, the most frequent reasons for the patient’s choices were religious beliefs and cultural traditions [6-8], with sex being the most important parameter among the very religious communities [6,7].

Israel is a multi-religious state that is composed of many subpopulations specifically, 75.6% Jewish, 16.9% Muslim, 2% Christians, 1.4% Druze and 4.1% others [16]. This variety provides the opportunity to conduct many investigations on the preferences of specific subpopulations within the country [10-13,17]. Two main female-sex evolutionary processes associated with medicine have taken place in Israeli society over the past few years. The first is an exponential increase in the percentage of women physicians: in 2009, 47% of the physicians under 45 were women compared to 27.5% in 1989 [18]. The second was a greater preference of women for female physicians, especially in the field of obstetrics and gynecology, driven by modern secular feminists [13] as well as by religious/cultural influences [11,12].

The Druze are part of the Arab minority in Israel. The Druze culture is Arab and their language Arabic. They comprise a closed, monotheistic religious community, which emerged during the 11th century from Islam. The Druze faith began as a movement in Islam that was mainly influenced by Greek philosophy and Gnosticism opposing certain religious and philosophical ideologies that were present during that epoch.

Most of the Druze live in Syria (40%–50%), some in Lebanon (30%–40%), a smaller fraction in Israel (6%–7%) and the rest in Jordan (1%–2%). The Israeli Druze community numbers around 140,000, most of whom live in villages in the northern part of the country [19]. In 1957, at the request of its communal leaders, the Israeli government designated the Druze as being a distinct ethnic community, and an autonomous religious community, independent of Muslim religious courts. Members of the community have attained top positions in Israeli politics and public service [19-22].

The Druze community is divided into two major groups: 80% called *al-Juhhāl* who are divided into 3 sub-groups: traditional, religious, and secular; and the other 20% who are ultra-religious (called *al-Uqqāl-*“the Knowledgeable Initiates”). Males and females of the latter group dress according to tradition and modesty codes [19].

According to Druze religious law, the women are granted status nearly equal to that of men. A Druze woman has the right to separate from and divorce her husband without his agreement, she can inherit her parent’s property, and she can be a priestess. In practice, like other traditional Arab societies, the Druze community is conservative when it comes to social relations, marriage connections, and women’s rights. Traditionally, Druze women mainly attended to housework and child-rearing, and were generally subservient to males. Druze rules regarding a woman’s modesty (*al-mandīl*) are strictly defined. With the exception of first-degree relatives, males are generally not permitted to look at or touch women. Accordingly, women are not allowed to be examined by male physicians. Only during life-threatening emergencies when a female physician is not available a woman can be treated by a male physician. Consequently, large numbers of Druze women avoid seeking medical attention for gynecologic conditions for fear of being exposed to male physicians [19-22]. But, the status of Druze women has undergone a silent revolution over the last 20 years, and their position has been transformed through higher education: there are currently more Druze women than men in higher education and many limiting norms affecting Druze women have undergone considerable changes [19-22].

Similar studies that were conducted among other traditional Arab religious populations demonstrated the importance of sex when choosing obstetrician/gynecologist (6,7,11). Amir et al. found a 76.6% preference rate of Muslim-Arab women for female obstetricians/gynecologists [11]. Likewise, Rizk et al., found an 86.4% preference of responders from United Arab Emirates [6], and Lafta et al. reported that 73% of Iraqi female patients preferred a female obstetrician/gynecologist [7]. The objective of this study was to explore the sex preference of Israeli Druze women regarding their choice of obstetricians/gynecologists. Additional relevant characteristics affecting these choices were examined as well. Due to the very sparse information on the Druze community in general, and their specific needs from the health system in particular, this survey is timely and important insofar as it might also support modifications in the local health system.

## Methods

In light of the anticipated difficulty in collecting information from Druze women, we chose to perform the study in health centers located in five Druze villages in Northern Israel: Julis, Abu-Snan, Yarka, Daliah, and Bet-Jan. These villages contain a predominantly Druze population, and therefore provide services mainly to the Druze inhabitants. The study was conducted over a 6-month period in 2011 and approved by the Tel Aviv Sourasky Medical Center’s Institutional Review Board. An anonymous questionnaire written in Hebrew was used to assess Israeli Druze women’s preferences in selecting their obstetrician/gynecologist.

These five community health centers provide every type of ambulatory gynecological/obstetrical care for women. The staff consists of board certified obstetricians/gynecologists, nurses and ultrasound technicians. Residents who belong to the nationwide health-care organization (HMO) are entitled to the services of these centers. The questionnaires were distributed by the obstetricians/gynecologists in each community health center who provided detailed explanations about the study and the questionnaire. Five gynecologists in total participated in the distribution of questionnaires, four females and one male. One female gynecologist is Druze, whereas the other doctors do not share a common cultural or religious background with the women. From our experience in other studies carried out among minority populations, especially with religious affiliation, it is much easier to recruit people to participate in a study without the need of the participants to identify themselves in any way. Therefore, only oral consent to participate was obtained in order to preserve anonymity and thereby increasing the number of women who agreed to participate in the study. The women who agreed to participate filled out a self-administered, anonymous questionnaire in a secluded room and deposited the form in a designated box. The criteria used for participation in the study was that the women were at least 18 years old.

After collecting a total of 210 questionnaires, a similar number of questionnaires were analyzed in previous studies that we conducted among minority populations, the recruitment process was stopped. Of the 210 eligible women, 14 had declined to participate, and the remaining 196 were included in the final analysis.

We developed a questionnaire that incorporated items from previously validated instruments that assessed sex preference of women for their obstetricians and gynecologists [6,10-12,23]. Two major changes were made: demographic questions were adjusted to the population, and physician’s procedures were adapted to obstetric/genecology field. We conducted a pilot study among 20 Druze women and slightly revised the questionnaire before it was distributed to all the study participants. The final questionnaire was comprised of 36 items. The first part of the questionnaire concerned basic socio-demographic information, including age, country of origin (Israel/other), religion (Jewish/Christian/Muslim/Druze/other), religious status (religious/secular), everyday language (Arabic/Hebrew/other), knowing Hebrew (yes/no), marital status (single/in union/married/divorced/widow), children (yes/no), number of children, education (primary school/high school/college/university), employment status (working/not working), and the sex of her regular obstetrician/gynecologist (regular obstetrician/gynecologist was defined as obstetrician/gynecologist who the woman visited in the last three years) (male/female). Importantly, the religious status of the responders was classified into one of two groups: secular versus religious. The classification was measured according to their self-definition and estimation of being secular versus religious. Although the religious group contains ultra-religious, religious and traditional, we did not distinguish between those subgroups in the current study. The items in the first part were answered by circling the appropriate option. The second part included questions about sex preferences for obstetricians/gynecologists when they required a gynecological examination, pregnancy follow-up, cesarean section, gynecologic surgery, or any consultation for an obstetric or gynecologic problem. The women were asked about sex bias associated with specific obstetric/gynecologic procedures, such as embarrassment or comfort during a gynecological examination. They were also asked to identify specific characteristics of obstetrician/gynecologist as related to their sex. These included being gentle, sympathetic, patient, spends more time with patient, understanding in women’s health, knowledgeable in women’s health, and better physician in general. The participants answered these questions by circling the word “male”, “female” or “none” next to each characteristic. Sex preference was scored 0 for male, 1 for female and 2 for none. Each participant was also asked to circle three out of a list of 16 characteristics (listed in Table 1) she considered to be the most important in choosing her obstetrician/gynecologist.

**Table 1:** **List and percentage of the 16 factors ranked by women affecting their choice for a gynecologist/obstetrician**

**Factors**	**Number (%)**
Demographics	
Age	17 (8.7)
Gender	61 (31.1)
Origin	13 (6.6)
Marital status	5 (2.6)
Parental status	1 (0.5)
Religious status	4 (2)
Professional skills	
Ability (professional)	177 (90.3)
Experience	180 (91.8)
Knowledge	166 (84.7)
Qualifications	
Board certification	105 (53.6)
Schools attended	19 (9.7)
Hospital affiliation	44 (22.4)
University affiliation	6 (3.1)
Other qualities	
Personality	60 (30.6)
Reputation	71 (36.4)
Availability	92 (46.9)

Descriptive statistics are given as mean for continuous variables and frequency distribution for categorical variables. A Chi square test (for categorical variable) and *t*-test (for continues variables) were applied to examine the relationship between the women’s demographic characterization and gynecologist sex preference, and between the physician’s characterization and gynecologist sex preference.

The McNemar test for symmetry (which enables a paired comparison instead of a group comparison) was used to compare preference for physician’s sex with regard to obstetricians/gynecologists vs. family physician, to compare obstetricians/gynecologists sex preference for intimate procedures vs. non-intimate procedures, and to compare obstetricians/gynecologists sex preference with regard to different physician characteristics (embarrassment, patience, spends more time with patient, understanding in women’s health, knowledgeable in women’s health and better physician in general).

A multiple logistic regression was applied in order to assess which variables related to women or physician, were independently associated with preference for a female gynecologist. Preference for gynecologist’s sex was coded as 1 for indifference/male preference, and 2 for female. The model predicts the probability of having the value 2 (the preference for a female gynecologist). The reference category for religious status was 0 = secular, for marital status was 0 = non-married, for children was 1 = with children, for education was 4 = university, and for employment status was 1 = working. Each responder was asked to rank how important sixteen different variables are in relation to her preference of physician’s sex. Variables were coded on a scale of 0–3, with 0 being no preference, 3 for highest preference. Variables were then combined into four categories by clustering all of the variables in each category: demographics, professional skills, qualifications and other qualities. Physician’s sex variable is clearly associated with preference for gynecologist’s sex, and therefore, was eliminated from demographic category. All statistical analyses were performed using the SAS for Windows 9.2.

## Results

The study sample was comprised of 196 eligible women whose demographic and other characteristics are presented in Table 2. The mean age of the sample was 34 years old. Sixty (30.9%) of the responders were secular and one hundred thirty four (69.1%) were religious. Thirty (15.3%) were not married and one hundred sixty six (84.7%) were married. One hundred sixty (81.6%) had children and thirty six (18.4%) didn’t have children. Regarding education: twenty one (10.7%) finished primary school, thirty seven (18.9%) high school, twenty nine (14.8%) college and one hundred and nine (55.6%) university. One hundred sixty four (83.7%) were employed and thirty two (16.3%) were un-employed. Forty six (23.5%) had a regular male obstetricians/gynecologists in the last 3 years, while one hundred and fifty (76.5%) had a female obstetricians/gynecologists.

**Table 2:** **Relations between characteristics of the women and the gender preference of obstetricians/gynecologists**

**Characteristic**	**Total participants**	**Prefer woman Ob/Gyn**	**Prefer man Ob/Gyn or No sex preference for Ob/Gyn**	***P*** **value**
Age (y), mean (range)	34 (19–70)	35.3	32.7	0.0316
Religious status				0.0232
Secular	60 (30.9%)	31 (25.2%)	29 (40.85%)	
Religious	134 (69.1%)	92 (74.8%)	42 (59.15%)	
Marital status				NS
Non-married	30 (15.3%)	22 (17.6%)	8 (11.27%)	
Married	166 (84.7%)	103 (82.4%)	63 (88.73%)	
Children				NS
Yes	160 (81.6%)	99 (79.2%)	61 (85.92%)	
No	36 (18.4%)	26 (20.8%)	10 (14.08%)	
Education				NS
Primary school	21 (10.7%)	14 (11.2%)	7 (9.86%)	
High school	37 (18.9%)	20 (16%)	17 (23.94%)	
College	29 (14.8%)	19 (15.2%)	10 (14.08%)	
University	109 (55.6%)	72 (57.6%)	37 (52.11%)	
Employment				NS
Yes	164 (83.7%)	102 (81.6%)	62 (87.32%)	
No	32 (16.3%)	23 (18.4%)	9 (12.68%)	
Sex of the regular				<.0001
Ob/Gyn				
(last three years)				
Male	46 (23.5%)	11 (8.8%)	35 (80%)	
Female	150 (76.5%)	114 (91.2%)	36 (20%)	

Table 3 displays the responder’s sex preferences for obstetricians/gynecologists and for family physicians: the preference for female obstetricians/gynecologists was most conspicuous. There was a significantly higher preference for female obstetricians/gynecologists than female family physicians (McNemar test = 88; *p* < 0.001). Interestingly, when exploring sex preferences for obstetricians/gynecologists among sub-populations of the responders, there was a significant preference for female obstetricians/gynecologists among the religious compared to the secular women (*X*^2^ = 5.1557; *p* = 0.0232) (Table 4).

**Table 3:** **Gender preferences of family physicians and obstetricians/gynecologists of 196 female Druze responders**

	**Obstetrician/Gynecologist (%)**	**Family physician (%)**
Prefer male physician	5 (2.5)	13 (6.6)
Prefer female physician	125 (63.8)	37 (18.9)
No preference	66 (33.7)	146 (74.5)

**Table 4:** **Gender preferences of 196 female Druze responders for family physicians and obstetricians/gynecologists in relation to religious status**

	**Religious** ***n*** **(%)**	**Secular** ***n*** **(%)**
Family physician		
Prefer male physician	12 (8.9)	1 (1.6)
Prefer female physician	26 (19.4)	11 (18.3)
No preference	96 (71.6)	48 (80)
Obstetrician/Gynecologist		
Prefer male physician	3 (2.2)	2 (3.3)
Prefer female physician	92 (68.6)	31 (51.67)
No preference	39 (29.1)	27 (45)

Three characteristics were associated with sex preference for female obstetricians/gynecologists: the age and religious status of the woman, and the sex of her regular obstetrician/gynecologist. Respondents who preferred female obstetricians/gynecologists were older (p = 0.0316), more religious (p = 0.0232), and their regular obstetricians/gynecologists were female (p < 0.0001) (Table 2).

Even though the majority of the surveyed women (65.3%) preferred to undergo a pelvic examination and 61.9% preferred pregnancy follow-up by a female obstetrician/gynecologist, procedures that are considered “intimate”, only the minority preferred female obstetricians/gynecologists, when it came to non-intimate procedures (Table 5). For example, although there was a significant preference to undergo a pelvic examination by a female obstetricians/gynecologists, there was not for a cesarean section (McNemar test = 63.48; *p* < 0.001), gynecologic surgery (McNemar test = 39.7241; *p* < 0.001), or advice for a major obstetric/gynecologic problem (McNemar test = 35.5; *p* < 0.001). In addition, there was a significant preference to undergo pregnancy follow-up by a female obstetricians/gynecologists but not for a cesarean section (McNemar test = 59.2; *p* < 0.001), gynecologic surgery (McNemar test = 29.49; *p* < 0.001), or advice for a major obstetric/gynecologic problem (McNemar test = 26.7; *p* < 0.001).

**Table 5:** **Druze women’s gender preference for obstetricians/gynecologists by intimate vs. non-intimate procedure performed**

	**Female (%)**	**No preference (%)**	**Male (%)**
Intimate procedure			
Pelvic examination	128 (65.3)	67 (34.2)	1 (0.5)
Pregnancy follow-up	120 (61.9)	68 (35.1)	6 (3.1)
Non-intimate procedure			
Cesarean section	57 (29.4)	123 (63.4)	14 (7.2)
Gynecologic surgery	78 (40.2)	104 (53.6)	12 (6.2)
Advice for major OB/GYN problem	82 (42.1)	96 (49.2)	17 (8.7)

p < 0.001 for Pelvic examination performed by female Ob/Gyn vs. Cesarean section, Gynecologic surgery and Advice for major OB/GYN problem performed by female Ob/Gyn.

p < 0.001 for Pregnancy follow-up performed by female Ob/Gyn vs. Cesarean section, Gynecologic surgery and Advice for major OB/GYN problem performed by female Ob/Gyn.

Feeling more comfortable and less embarrassed during intimate procedures were given as the main cause of female obstetricians/gynecologists preference (Table 6). Only a very small fraction of responders felt embarrassed during intimate procedure with female obstetricians/gynecologists (3.8%), while 69.7% felt more comfortable with female obstetricians/gynecologists and 56.6% thought that women obstetricians/gynecologists were gentler.

**Table 6:** **Druze women’s gender preference for obstetricians/gynecologists by perceived physician characteristics**

	**Female** ***n*** **, (%)**	**No preference** ***n*** **, (%)**	**Male** ***n*** **(%)**
Feeling during pelvic examination			
More embarrassment	7 (3.8)	94 (50.8)	84 (45.4)
More comfortable	136 (69.7)	55 (28.2)	4 (2.1)
More gentle	111 (56.6)	77 (39.3)	8 (4.1)
Physician’s characteristics			
More sympathetic	71 (37.2)	106 (55.5)	14 (7.3)
More patient	59 (30.6)	108 (56)	26 (13.5)
Spends more time with patient	67 (34.7)	114 (59.1)	12 (6.2)
Physician’s professionalism			
More understanding in women’s health	55 (28.2)	133 (68.2)	7 (3.6)
More knowledgeable in women’s health	47 (24.1)	134 (68.7)	14 (7.2)
Better physician in general	41 (21.2)	139 (72)	13 (6.7)

*p* < 0.001 for More embarrassment during pelvic examination with male Ob/Gyn vs. More sympathetic, More patient and Spends more time with patient, More understanding in women’s health, More knowledgeable in women's health and Better physician in general, as a reasons for female Ob/Gyn preference.

Most of the patients answered that they have no preference with respect to physician’s personality (sympathetic, patient, spending time with patient) and professionalism (understanding of women’s health, knowledgeable in women’s health, general physician skills). There was a significant preference for choosing a female obstetricians/gynecologists due to embarrassment with male obstetricians/gynecologists rather than due to a physician’s characteristics, such as being more sympathetic (McNemar test = 57.2462; *p* < 0.001), more patient (McNemar test = 46.2963; *p* < 0.001)), and spending more time with the patient (McNemar test = 48.0159; *p* < 0.001). In addition, there was a significant preference for choosing a female obstetricians/gynecologists due to embarrassment with male obstetricians/gynecologists rather than due to physician’s professionalism, e.g., more understanding in women’s health (McNemar test = 39.1852; *p* < 0.001), more knowledgeable in women’s health (McNemar test = 30.0833; *p* < 0.001), and being a better physician in general (McNemar test = 28.4444; *p* < 0.001).

The participants ranked experience (91.8%), ability (90.3%) and knowledge (84.7%) as the top three qualities in an obstetrician/gynecologist (Table 1). Other characteristics, such as demographic background, qualifications and other selected qualities (personality, reputation and availability) were less important. Two physician’s factors were associated with sex preference for female obstetricians/gynecologists: sex, and knowledge. Physicians’ sex was important to responders who preferred female obstetricians/gynecologists (p < 0.0001), while physicians’ knowledge was less important to these responders who preferred female obstetricians/gynecologists (p < 0.0046) (Table 7).

**Table 7:** **Relations between characteristics of the physician and the gender preference of obstetricians/gynecologists**

**Characteristic**	**Prefer women Ob/Gyn**	**Prefer man Ob/Gyn and no sex preference for Ob/Gyn**	***P*** **value**
Demographics			
Age			NS
Selected	12 (9.6%)	5 (7.04%)	
Not selected	113 (90.4%)	66 (92.96%)	
Gender			<.0001
Selected	58 (46.4%)	3 (4.23%)	
Not selected	67 (53.6%)	68 (95.77%)	
Origin			NS
Selected	8 (6.4%)	5 (7.04%)	
Not selected	117 (93.6%)	66 (92.96%)	
Marital status			NS
Selected	3 (2.4%)	2 (2.82%)	
Not selected	122 (97.6%)	69 (97.18%)	
Parental status			NS
Selected	1 (0.8%)	0 (0%)	
Not selected	124 (99.2%)	71 (100%)	
Religious status			NS
Selected	1 (0.8%)	3 (4.23%)	
Not selected	124 (99.2%)	68 (95.77%)	
Professional skills			
Ability			NS
Selected	111 (88.8%)	66 (92.96%)	
Not selected	14 (11.2%)	5 (7.04%)	
Experience			NS
Selected	112 (89.6%)	68 (95.77%)	
Not selected	13 (10.4%)	3 (4.23%)	
Knowledge			0.0046
Selected	99 (79.2%)	67 (94.37%)	
Not selected	26 (20.8%)	4 (5.63%)	
Qualifications			
Board certification			NS
Selected	63 (50.4%)	42 (59.15%)	
Not selected	62 (49.6%)	29 (40.85%)	
Schools attended			NS
Selected	14 (11.2%)	5 (7.04%)	
Not selected	111 (88.8%)	66 (92.96%)	
Hospital affiliation			NS
Selected	27 (21.6%)	17 (23.94%)	
Not selected	98 (78.4%)	54 (76.06%)	
University affiliation			NS
Selected	4 (3.2%)	2 (2.82%)	
Not Selected	121 (96.8%)	69 (97.18%)	
Other qualities			
Personality			NS
Selected	37 (29.6%)	23 (32.39%)	
Not selected	88 (70.4%)	48 (67.61%)	
Reputation			NS
Selected	46 (36.8%)	25 (35.71%)	
Not selected	79 (63.2%)	45 (64.29%)	
Availability			NS
Selected	54 (43.2%)	38 (53.53%)	
Not selected	71 (56.8%)	33 (46.47%)	

Lastly, in multiple logistic regression analysis we found independent predictors for choosing female obstetricians/gynecologists: older, and religious responders in addition to women who assigned a lesser weight to the professional level of obstetricians/gynecologists were more likely to prefer female obstetricians/gynecologists (Table 8).

**Table 8:** **Multiple logistic regression analysis to estimate the variables that were significant in preference for a female gynecologist**

**Parameter**	**Coefficient**	**Pr > ChiSq**	**Odds ratio**	**95% Wald**	
				**Confidence limits**	
Age	0.0654	0.0093	1.068	1.016	1.122
Religious status	0.6891	0.0451	1.992	0.988	4.016
Marital status	-0.2467	0.6553	0.781	0.265	2.308
Children	0.9709	0.0772	2.640	0.900	7.749
Education_1	-0.5429	0.2536	0.275	0.075	1.010
(Primary school)					
Education_2	-0.3447	0.3048	0.335	0.137	0.818
(High school)					
Education_3	0.1389	0.7189	0.543	0.197	1.496
(College)					
Employment	0.8204	0.0936	2.271	0.871	5.926
Demographics	0.3796	0.8286	1.462	0.047	45.421
Professional skills	2.6802	0.0091	14.588	1.948	109.247
Qualifications	-0.3119	0.7485	0.732	0.109	4.927
Other qualities	0.3307	0.5406	1.392	0.483	4.015

## Discussion

Modern medicine is beginning to recognize the importance of the perspective of the patient in health care, emphasizing the importance of inter-relationships of health needs, satisfaction and quality of life. Many studies have been conducted on patient’s preference of their obstetrician/gynecologist, but the majority of them were conducted in modern western societies, establishing a need to explore other populations. The present study was performed in the Israeli Druze community which is composed of a unique population that practices centuries-old traditions. To the best of our knowledge, this is one of a very few studies that were conducted among them.

In general, two factors consisting of feminization among modern populations [3,4,13] and traditional beliefs among religious populations [6,7,11,12] have led to same-sex preference of obstetricians/gynecologists. Interestingly, contrary findings have been reported also among women in modern western societies [10,23]. Due to the conservative nature of the Druze community, it did not come as any surprise that most of our Druze religious and non-religious responders (63.8%) preferred a female obstetricians/gynecologists.

We found 3 parameters in responder’s characteristics that correlates to the preference of female obstetricians/gynecologists, among them religiousness of the responders, which is also predictive parameter for choosing female obstetricians/gynecologists. The results emphasize again the importance of the religiousness as a factor in the decision of the Druze women regarding their preference of their obstetricians’/gynecologists’ sex, similar to other religious and traditional populations [6-8,11,12].

Still, looking at the percentage of same sex preference among other Arab traditional-populations, the percentage of Druze responders who preferred female obstetricians/gynecologists were lower than in the United Arab Emirates [6], Israeli Muslim Arabs [11], and Iraqi responders [7]. This was unexpected due to the similar religious and tradition nature of the Druze population. The differences might result from the higher percentage of secularity among our Druze responders compared to the secularity percentage among other Arab religious populations like the Israeli Muslim Arabs [11], which probably influence the preferences.

We also found significant differences between the religious Druze fraction and the secular one, regarding obstetricians/gynecologists sex preference. In a similar manner, secular Jewish responders significantly differ from religious Jews regarding physician sex preference [12]. However comparison of the religious Druze to their Jewish counterpart found that only 68.6% of the religious Druze women preferred a same-sex physician compared to 92.9% of the religious Jewish responders [12]. This discrepancy might stem from differences in the status of religiousness; in the case of Jewish responders the religious were only ultra-orthodox while in the Druze responders the religious are composed of ultraorthodox, religious and traditional responders.

An additional parameter that was correlated to female preference and is a predictive parameter is age of the responders. Other papers demonstrated that age is important to sex preference, including Makam et al. [24], and Lafta et al. [7]. However the latter demonstrated an opposite association; as age increased there was a decrease in female preference. Their explanation was that the life experiences of the older responders help them to see other factors as more important [7]. For the past two decades, many young Druze women have been seeking higher education and have become more integrated into the western/modern society. The integration and education have led to less religious and tradition adherence among the young Druze women [19-22,25]. Since there is no association between Druze responders education or employment status to obstetricians/gynecologists sex preference, we believe that age is the important factor, since older Druze women tend to be more religious and adhere more to tradition therefore choosing female obstetricians/gynecologists [19-22,25].

A patient’s preference for same-sex physicians is more evident in more intrusive fields, such as obstetrics and gynecology, compared to family physician [8,11,12]. Indeed, as had been seen in other populations, the Druze women we queried exhibited same-sex physician preference for their obstetrician/gynecologist (63.8%) compared to their family physicians (18.9%). Similarly, Druze women also exhibited same-sex physician preference for other intimate procedures, such as colonoscopy [6,8,11,12,17,23-26].

Furthermore, the main reasons for preferring female obstetricians/gynecologists by Druze responders were feeling more comfortable and less embarrassed, and the notion that female obstetricians/gynecologists are more gentle during intimate procedures. Others studies conducted among Arab traditional and religious populations found that one of the main reasons for sex preference is feeling more comfortable and less embarrassed when being treated by female obstetricians/gynecologists [7,12], and specifically for intimate procedures [12].

The sex of the regular obstetricians/gynecologists of the responders is the third parameter correlated to female obstetricians/gynecologists preference. This finding is supported by Piper et al. [10] and Schmittdiel et al. [27] who both demonstrated that the sex of the regular obstetricians/gynecologists influence the decision of obstetricians/gynecologists sex. We assume that preference of same sex obstetricians/gynecologists is influenced from positive experience with the regular obstetricians/gynecologists (though we don’t have data). Indeed when you choose only the responders with positive experience from their regular male/female responders, you eliminate sex preference for female [28].

The top three characteristics chosen by our Druze study participants applied to the obstetrician’s/gynecologist’s professional skills (i.e., experience, knowledge, and ability). Importantly, despite the top-rankings of professional skills, we found that women who preferred a female obstetricians/gynecologists assigned a lesser weight to knowledge in choosing a gynecologist. Moreover, we found that women who attributed reduced weight to professional level of obstetricians/gynecologists can be predicted to prefer female obstetricians/gynecologists. Previous studies found that among modern and western communities physician’s professionalism were important and associated to obstetricians/gynecologists preference rather than sex [3,10], while among religious Muslim other factors were more important, specifically sex [6,7]. Druze responders, like other religious communities, display association to sex rather than professional skills. Still, even the fraction of Druze women who preferred female obstetricians/gynecologists ranked with high percentage the preference for professional skills (~80% and above), suggesting that their proximity and interaction with the Israeli secular western society have considerable influence on the way they choose their obstetricians/gynecologists. Notably, we do not have detailed information about the integration and influence of the Israeli modern society on the Druze- additional research is required to investigate this issue.

It should be noted that our study has several limitations. One limitation is our use of a new non-validated questionnaire since there was such a paucity of studies similar to ours. We developed a 36-item questionnaire that incorporated items from previously validated instruments to assess sex preference of an obstetrician/gynecologist. Another caveat is the fact that the questionnaire was in Hebrew and this could affect the ability of the responders, specifically those who are older and those who are less educated, to fully understand the questions. The third limitation is the fact that most of the regular obstetricians/gynecologists of the responders are female. We do not have much data regarding prior experience with their regular obstetricians/gynecologists. And, the high percentage of female obstetricians/gynecologists could contribute to the preference of female obstetricians/gynecologists. Another limitation is the fact that we did not gather information regarding other considerations that the woman might have when she chooses a particular physician (for example, family relationship with the physician, or the location of their physician inside or outside their village). The last limitation is our studying only Druze women within Israel’s borders and exposed to the Israeli modern western lifestyle. It would be interesting to explore additional Druze populations from other countries that we expect are less exposed to western influences.

## Conclusion

The Druze community is one in which tradition and religion play pivotal roles. Altogether the Druze community, like other communities where religiousness is deeply rooted, prefer female obstetricians/gynecologists. The main reasons, like in other Arab communities, are feeling more comfortable and less embarrassed with females, and the notion that female obstetricians/gynecologists are more gentle during intimate procedures, that are connected to the modesty codes and norms of the religious Arab populations. The preference of the Druze responders was associated to responder’s religiousness and age, as well as the sex of her regular obstetrician/gynecologist. Additionally, women who preferred a female obstetricians/gynecologists assigned a lesser weight to knowledge in choosing a gynecologist. Predictively, older and religious women are more likely to prefer a female obstetrician/gynecologist as well as women who attribute reduced weight to professional level in choosing an obstetrician/gynecologist.

Given the current surge of women entering medical practice over the past few decades, we expect that Druze women will be able to find exactly what they prefer in the near future.
